# Do COVID-19 Vaccination Policies Backfire? The Effects of Mandates, Vaccination Passports, and Financial Incentives on COVID-19 Vaccination

**DOI:** 10.1177/17456916231178708

**Published:** 2023-12-04

**Authors:** Bita Fayaz-Farkhad, Haesung Jung

**Affiliations:** Annenberg School for Communication, University of Pennsylvania

**Keywords:** COVID-19 vaccination, policy, mandate, vaccination passport, financial incentive

## Abstract

Faced with the challenges of motivating people to vaccinate, many countries have introduced policy-level interventions to encourage vaccination against COVID-19. For example, mandates were widely imposed requiring individuals to vaccinate to work and attend school, and vaccination passports required individuals to show proof of vaccination to travel and access public spaces and events. Furthermore, some countries also began offering financial incentives for getting vaccinated. One major criticism of these policies was the possibility that they would produce reactance and thus undermine voluntary vaccination. This article therefore reviews relevant empirical evidence to examine whether this is indeed the case. Specifically, we devote separate sections to reviewing and discussing the impacts of three major policies that were implemented during the COVID-19 pandemic: vaccination mandates, vaccination passports, and the provision of financial incentives. A careful analysis of the evidence provides little support that these policies backfire but instead can effectively promote vaccination at the population level. The policies are not without limitations, however, such as their inability to mobilize those that are strongly hesitant to vaccines. Finally, we discuss how policy-level interventions should be designed and implemented to address future epidemics and pandemics.

Despite the availability of COVID-19 vaccines, which have been widely distributed since early 2021, many countries are still struggling to push vaccination rates beyond 80%. As of October 2022, the rate of fully vaccinated Americans is 68%, and rates of other high-income countries range from 76% (Germany, United Kingdom) to 79% (France; Holder, 2023). To make matters worse, the rate of newly vaccinated individuals continues to decline (Ramos, 2022), and when considering the rate of those who received an additional dose, the rates plummet, particularly in the United States (34% of individuals received at least one additional dose; Holder, 2023). The difficulties associated with getting people to vaccinate pose a significant threat to public health as new variations of COVID-19 continue to evolve, and they are almost impossible to control without motivating people to vaccinate and vaccinate regularly.

To encourage vaccination, many countries intervened at the policy level. For example, policies mandating vaccination against COVID-19 imposed by government and private employers are among the most controversial but were widely implemented to control the spread of COVID-19. As of May 2022, about four in 10 employers in the United States had some form of COVID-19 vaccine mandate (Iafolla, 2022). Vaccine passports were also adopted by dozens of countries that required people to present their proof of receiving at least two doses of COVID-19 vaccine to travel internationally. Some countries have also used them domestically to grant access to shared public places and events such as nightclubs, hospitals, gyms, and concerts. Israel began implementing the passport policy in early 2021 (Rouw et al., 2021), and other countries, including Denmark, France, Germany, Italy, and Qatar, as well as several Canadian provinces and the state of New York, have issued similar policies. Some countries adjusted these restrictions over time as the pandemic progressed (Drew, 2022). Last, programs that offer financial incentives ranging from small, guaranteed rewards (e.g., gift cards, free food) to lotteries in which people could win a large sum of money after getting vaccinated were introduced. In the United States, Ohio was one of the first states to begin incentive programs that offered opportunities to win $1 million among vaccinated adults in addition to full academic scholarships among vaccinated teens. This was followed by 23 more states implementing similar incentive programs by the end of 2021. Other countries, including China, the United Kingdom, Germany, Russia, and Australia, have implemented equivalent incentive policies to encourage vaccination (Elkes, 2021).

Such policies typically adopt behaviorist principles to shape human behavior (Watson, 1913, 1998) by pairing vaccination with positive outcomes (e.g., providing an incentive when one chooses to vaccinate) and nonvaccination with negative outcomes (e.g., restricting one’s ability to work or travel when one chooses not to vaccinate). Assuming the valence of behavioral outcomes is the only thing that governs human behavior, the policies should be effective in increasing vaccination.

Nonetheless, the efficacy of such policies has been widely debated for several reasons. Because such vaccine policies essentially represent external forces to shape behavior, some studies have argued that their implementation can produce psychological reactance, a negative emotional response caused by threats to or losses of behavioral freedom (J. Brehm, 1966, 1972; S. S. Brehm & Brehm, 2013). Specifically, people generally believe that they possess certain levels of freedom and desire to manifest it by having control over their own choices and courses of actions. However, when they encounter events that threaten or restrict their sense of freedom, they become motivated to restore it by acting against these events. In the context of vaccination then, when people feel that relevant policies restrict their freedom to choose whether to vaccinate, this in turn could lead to the unintended consequences of reducing their vaccination intentions and/or uptake (Sprengholz, Felgendreff, et al., 2022). Policies such as vaccination mandates and passports have a greater chance of eliciting psychological reactance because these policies have direct control over what people can and cannot do depending on their vaccination status. Relatedly, external forces to vaccinate may also undermine intrinsic motivation, such as vaccinating to protect one’s community (Largent & Miller, 2021; Schmelz, 2020). Some have also argued that the introduction of COVID-19 vaccination policies can give the impression that people would not vaccinate otherwise and therefore ironically signal that COVID-19 vaccines are unsafe and undesirable (Savulescu, 2021). Others have further argued that the implementation of such policies are too simplistic and shortsighted and thus do not fully address the fundamental challenges to vaccination, such as access to health care and vaccine hesitancy (Volpp & Cannuscio, 2021).

Given the mixed sentiments surrounding the policy-level attempts to motivate vaccination, numerous works have empirically examined their impacts on vaccination. In this article, we review and discuss the effects of three representative COVID-19 vaccination policies on vaccination outcomes: mandates, vaccination passports, and financial incentives. In doing so, we primarily focus on elucidating their effects at the population level because most policies are implemented this way. We nonetheless carefully review variables that may strengthen or weaken the effects to inform how policies may yield divergent effects across subpopulations and contexts. Overall, our review of relevant studies provides little evidence that COVID-19 vaccination policies negatively affect vaccination outcomes at the population level. However, the evidence suggests that numerous factors contribute to the heterogeneity of the effect, including policy characteristics such as the delay between the policy announcement and implementation and demographic characteristics such as partisanship and income level. The evidence further suggests that some policies are more likely to have only a short-term impact, and they may be less effective in persuading strongly hesitant people to vaccinate. Our findings confirm the general expectation that vaccination policies have a lesser impact in regions with high vaccination rates in the first place, producing regional-level variations in their effectiveness. Last, several studies in our review suggest that the effects of policies diverge depending on whether the studies measured vaccination intentions or behavior and whether the studies measured first-dose vaccination or full vaccination as their primary outcome. We end by discussing several implications for designing policy-level interventions to encourage vaccinations in future epidemics and pandemics.

## Article Search and Selection Criteria

We used a set of predetermined criteria to select articles to review. Specifically, we were interested in reviewing articles that quantitatively examined the effects of mandates, vaccination passports, and financial incentives on COVID-19 vaccination. Therefore, we used combinations of the following terms to search relevant published and unpublished articles on Google Scholar, PsycINFO, EBSCO, and PubMed: polic*, mandat*, requir*, pass*, certificate, financial, pay*, bonus, incentive*, lotter*, mone*, COVID-19, coronavirus, vaccination, vaccine, and booster. Our search was conducted between March and October 2022. We removed duplicates from our initial pool of articles and retained articles that (a) examined the effects of COVID-19 vaccination policies; (b) tested their effects on behavioral outcomes, including vaccination intentions and uptake; and (c) compared the policy effects between an intervention group or period and control group or period. Because we did not plan on conducting a formal meta-analysis of the effects of COVID-19 policies on vaccination, reporting statistics adequate to calculate standardized effect sizes (e.g., Cohen’s *d*) was not part of our inclusion criteria. Nevertheless, most of the articles reported some statistics that could help inform the magnitude of the effects found. A complete list of the articles reviewed in this article as well as their study characteristics and effects are summarized in Table 1.

**Table 1.** Direction and Magnitude of Associations Between Policy and COVID-19 Vaccination for Mandates, Vaccine Passports, and Financial Incentives

Reference	Region	Study design	Predictor	Vaccination outcome	Outcome type	Moderators	Net direction	Magnitude
**Mandate policies**								
Albarracin et al. (2021)	U.S.	Experimental	Requirement for work, school, travel	Vaccinate against COVID-19	Intention	—	Positive	26%; Cohen’s *d* = 0.10–0.17
Sprengholz et al. (2021)	U.S.	Experimental	Mandatory (vs. voluntary) COVID-19 vaccination policies	Avoidance of future COVID-19 vaccine	Intention	Vaccine hesitancy	Negative	−13.7%
Sprengholz et al. (2021)	U.S.	Experimental	Mandatory (vs. voluntary) COVID-19 vaccination policies	Vaccinate against chicken pox	Intention	Vaccine hesitancy, vaccine scarcity	Positive	9.4%
Sprengholz, Felgendreff, et al. (2022)	Germany	Experimental	Mandatory (vs. voluntary) COVID-19 vaccination policies	Vaccine against influenza	Intention	—	No effect	22.1%
Sprengholz, Felgendreff, et al. (2022)	U.S.	Experimental	Self-relevant (non-self-relevant) mandate	Avoidance of future COVID-19 vaccine	Intention	Attitude toward mandates, communication, reactance	Unknown	—
Sprengholz, Felgendreff, et al. (2022)	U.S.	Experimental	Self-relevant (non-self-relevant) mandate	Vaccine against influenza	Intention	Attitude toward mandates, communication, reactance	Unknown	—
Sprengholz et al. (2021)	Germany	Experimental	Mandatory COVID-19 vaccination of children	Vaccinate against chicken pox	Intention	Attitude toward mandates	Negative	—
Fishman et al. (2022)	U.S.	Experimental	Requirement for work, travel, eating out	Vaccinate against COVID-19	Intention	Political orientation	Positive	1.4–8.6%
Lee et al. (2022)	U.S.	Correlational	Requirement for health-care personnel	One or more doses of COVID-19 vaccine	Behavior	—	Positive	23.5%
**Passport policies**								
Okamoto et al. (2022)	Japan	Experimental	Vaccine passport easing public-health restrictions	Vaccine acceptance	Intention	Age	Positive	4–10%
Walkowiak et al. (2021)	Lithuania and Poland	Correlational	Strict COVID-19 passport	At least a single dose of COVID-19 vaccine	Behavior	—	Positive	12.3%
Karaivanov et al. (2022)	Canada	Quasi-experimental	Proof of vaccination to access public venues and nonessential businesses	First-dose uptake of COVID-19 vaccine	Behavior	Timing of announcement, percentage of unvaccinated	Positive	66%
Oliu-Barton et al. (2022)	France	Quasi-experimental	Announcement of COVID vaccination certificates	At least a single dose of COVID-19 vaccine	Behavior	Communication, requirement scope, timing of the introduction	Positive	24.2%
Oliu-Barton et al. (2022)	Germany	Quasi-experimental	Announcement of COVID vaccination certificates	At least a single dose of COVID-19 vaccine	Behavior	Communication, requirement scope, timing of the introduction	Positive	9.7%
Oliu-Barton et al. (2022)	Italy	Quasi-experimental	Announcement of COVID vaccination certificates	At least a single dose of COVID-19 vaccine	Behavior	Communication, requirement scope, timing of the introduction	Positive	6.2%
Mills & Rüttenauer (2022)	Denmark	Quasi-experimental	COVID-19 vaccination certification	Daily COVID-19 vaccine doses per 1 million	Behavior	Percentage unvaccinated, vaccine supply, age	No effect	11%
Mills & Rüttenauer (2022)	Israel	Quasi-experimental	COVID-19 vaccination certification	Daily COVID-19 vaccine doses per 1 million	Behavior	Percentage unvaccinated, vaccine supply, age	Positive	25%
Mills & Rüttenauer (2022)	Italy	Quasi-experimental	COVID-19 vaccination certification	Daily COVID-19 vaccine doses per 1 million	Behavior	Percentage unvaccinated, vaccine supply, age	Positive	14%
Mills & Rüttenauer (2022)	France	Quasi-experimental	COVID-19 vaccination certification	Daily COVID-19 vaccine doses per 1 million	Behavior	Percentage unvaccinated, vaccine supply, age	Positive	41%
Mills & Rüttenauer (2022)	Germany	Quasi-experimental	COVID-19 vaccination certification	Daily COVID-19 vaccine doses per 1 million	Behavior	Percentage unvaccinated, vaccine supply, age	No effect	11%
Mills & Rüttenauer (2022)	Switzerland	Quasi-experimental	COVID-19 vaccination certification	Daily COVID-19 vaccine doses per 1 million	Behavior	Percentage unvaccinated, vaccine supply, age	Positive	27%
**Financial-incentive policies**								
Campos-Mercade et al. (2021)	Sweden	Experimental	Guaranteed monetary reward	Vaccinate against COVID-19	Intention	—	Positive	5.8%
Klüver et al. (2021)	Germany	Experimental	Hypothetical guaranteed monetary reward	Vaccinate against COVID-19	Intention	Vaccine hesitancy, communication	Positive	2.5–5.5%
Sprengholz, Felgendreff et al. (2022)	Germany	Experimental	Hypothetical guaranteed monetary reward	Vaccinate against COVID-19	Intention	Legal incentive	Positive	16.5%
Fishman et al. (2022)	U.S.	Experimental	Hypothetical guaranteed monetary reward	Vaccinate against COVID-19	Intention	Political orientation, education	Positive	9.2–17.1%
Kreps et al. (2021)	U.S.	Experimental	Hypothetical guaranteed monetary reward	Vaccinate against COVID-19	Intention	—	No effect	−1%
Taber et al. (2021)	U.S.	Experimental	Hypothetical lottery	Vaccinate against COVID-19	Intention	Lottery structure, baseline willingness	No effect	η_*p*_^2^ = .000–.03
Robertson et al. (2021)	U.S.	Experimental	Hypothetical guaranteed monetary reward	Vaccinate against COVID-19	Intention	Race, political orientation	Positive	8%
Iyer et al. (2022)	U.S.	Experimental	Hypothetical guaranteed monetary reward	Vaccinate against COVID-19	Intention	Vaccine hesitancy	Positive	22.3%
Raman et al. (2022)	U.S.	Experimental	Hypothetical guaranteed monetary reward	Vaccinate against COVID-19	Intention	Structure, vaccination status	Positive	3.77–19.12%
Campos-Mercade et al. (2021)	Sweden	Experimental	Guaranteed monetary reward	First-dose uptake of COVID-19 vaccine	Behavior	—	Positive	4.7%
Georgiou et al. (2022)	U.S.	Correlational	Guaranteed monetary reward	Full vaccination against COVID-19	Behavior	Sex, race	Positive	13.7%
Wong et al. (2022)	U.S.	Quasi-experimental	Guaranteed monetary reward	First-dose uptake of COVID-19 vaccine	Behavior	—	Negative	−26.4%
Thirumurthy et al. (2022)	U.S.	Correlational	Guaranteed monetary reward/lottery	Daily COVID-19 vaccine doses per 100,000	Behavior	Political orientation	No effect	−27.8%
Jacobson et al. (2022)	U.S.	Experimental	Guaranteed monetary reward	First-dose uptake of COVID-19 vaccine	Behavior	Age, political orientation	No effect	−14.2%
Mallow et al. (2022)	U.S.	Correlational	Lottery	Average number of COVID-19 vaccines administered	Behavior	Income	Positive	17.6%
Fuller et al. (2022)	U.S.	Correlational	Lottery	First-dose uptake of COVID-19 vaccine	Behavior	—	Positive	0.1–1.98%
Fuller et al. (2022)	U.S.	Correlational	Lottery	Full vaccination against COVID-19	Behavior	Political orientation	No effect	−1.16–2.15%
Guo et al. (2022)	U.S.	Correlational	Guaranteed monetary reward	COVID-19 vaccination rate	Behavior	Income, education, structure, employment status, rate of BIPOC populations	Positive	223%
Guo et al. (2022)	U.S.	Correlational	Lottery	COVID-19 vaccination rate	Behavior	Income, education, structure, employment status, rate of BIPOC populations	Positive	134.3%
Chetty-Makkan et al. (2022)	South Africa	Quasi-experimental	Guaranteed monetary reward	First-dose uptake of COVID-19 vaccine	Behavior	—	Positive	7.15–12.01%
Acharya & Dhakal (2021)	U.S.	Correlational	Lottery	First-dose uptake of COVID-19 vaccine	Behavior	—	Positive	23.1%
Walkey et al. (2021)	U.S.	Correlational	Lottery	Daily new adult vaccination per 100,000	Behavior	—	No effect	—

Note: Net direction and magnitude concern the net effects of relevant policies. If studies did not describe the significant test of net effects, we indicated their effects as unknown. To gauge the magnitude of policy effects, we converted their findings into percentage change by subtracting the preintervention or no-intervention vaccination percentage (baseline) from the postintervention or intervention percentage and dividing this by the preintervention or no-intervention percentage. When information to calculate this number was not available, we checked the supplementary materials. When relevant information was not available in the main article or in the supplementary materials, we deemed that we did not have sufficient information to calculate magnitude. When percentage change was not applicable (e.g., experimental studies with Likert scales), we reported other statistics that could inform the magnitude of policy effects (Cohen’s *d*, η_*p*_^2^). We categorized studies as correlational if a study did not manipulate or control any variables. We categorized studies as quasi-experimental if a study manipulated a variable but did not implement random assignment. We categorized studies as experimental if a study manipulated a variable and implemented random assignment. We reported effects in separate rows if a single study reported separate results for qualitatively different outcomes (e.g., first dose, second dose). We also reported effects in separate rows if a single study reported separate outcomes for different samples (e.g., U.S., non-U.S.). If an article had several studies and they were conceptual replications of one another (e.g., same IVs independent and dependent variables), we reported their effects in one row. Last, in the “Moderators” column, we reported moderators that significantly influenced the effects of policies rather than all moderators tested in each article. BIPOC = Black, Indigenous, and people of color.

As seen in Table 1, the studies included in our review were broadly categorized into three designs: experimental, quasi-experimental, and correlational. We categorized studies as experimental if a study manipulated a variable (i.e., policy implementation) and used random assignment. We categorized studies as quasi-experimental if a study manipulated a variable but did not implement random assignment. We categorized studies as correlational if a study did not manipulate or control any variables.

## Effects of Vaccination Mandates

Many organizations and federal agencies have imposed mandates requiring employee vaccination against COVID-19 to control virus transmission, protect employee health, and thereby minimize economic losses. Although these mandates have been subject to legal challenges and some have been halted or delayed, the vaccination requirements were incorporated into several federal contracts and subcontracts, and many private-sector employers had made significant progress on their implementation plans (Ferranna et al., 2022). Whereas vaccine mandates for other infectious diseases exist in some settings (e.g., school, health-care workers), population-wide adult mandates are unprecedented. Despite the widespread implementation of mandates to promote COVID-19 vaccination, their net effect remains unknown. On the one hand, requiring vaccination can increase its uptake because people are confronted with some form of negative consequences if they do not (e.g., not able to work or travel). On the other hand, mandates can produce psychological reactance that negatively impacts vaccination uptake.

### Overall effects of vaccination mandates on COVID-19 vaccination and infections

As shown in Table 1, only one quasi-experimental study has studied the causal impact of vaccination mandates on vaccination uptake and found that the requirements are an effective strategy for increasing vaccination. In particular, Lee et al. (2022) found that employer-enforced vaccination mandates (vs. no mandates) were associated with higher and more equitable vaccination uptake across sociodemographic groups among health-care personnel. Ghaffarzadegan (2022) found that the introduction of vaccination mandates (vs. no mandates) in colleges and universities substantially decreased COVID-19 cases in institutions of higher education by an average of 1,473 per 100,000 students, consistent with the effectiveness of vaccination mandates. Rather than just looking at how vaccination mandates in colleges affected the student body, Acton et al. (2022) examined how college-enforced COVID-19 vaccination mandates (vs. no mandates) also influence the health of the surrounding community. They found that the mandates decreased new COVID-19 cases by 339 cases per 100,000 residents in the surrounding county and decreased new deaths by over five per 100,000 residents.

The experimental evidence that examined intentions only also found that mandates increased vaccination intentions. In particular, Albarracin et al. (2021) investigated whether making vaccination a requirement for work, attending school, or traveling relative to encouraging free choice in vaccination strengthens vaccination intentions and found that vaccination mandates are likely to be successful in promoting vaccination relative to either giving people freedom to choose or reminding them of the freedom the vaccine might confer.

### Vaccination mandates on subgroups

Whereas existing evidence suggests that mandates effectively increased total vaccination uptake and contributed to containing the spread of the virus, bipolar attitudes toward vaccination mandates have been reported in surveys from the United States, Germany, Finland, Britain, and France (Gagneux-Brunon et al., 2022; Graeber et al., 2021; Largent et al., 2020; Slotte et al., 2022; Sprengholz, Korn, et al., 2022; Stead et al., 2022). Evidence from the United States, sub-Saharan Africa, and Germany suggest that demographic characteristics, socioeconomic status, and partisanship were associated with the acceptance of mandate policies (Graeber et al., 2021; Largent et al., 2020; Makadzange et al., 2022). For example, older individuals and males endorsed vaccination mandates more than did younger participants and females (Sprengholz, Korn, et al., 2022). Likewise, Democrats were more likely than Republicans and independents to accept mandates (Largent et al., 2020). Beyond demographics and socioeconomic status, the acceptance of vaccination mandates correlated with the perceptions of vaccine safety and their effectiveness (Makadzange et al., 2022; Sprengholz, Felgendreff, et al., 2022) as well as trust in health authorities (Slotte et al., 2022).

Emerging evidence indicates that when there is low support for mandate policies, they may backfire. A longitudinal study conducted in Germany during the COVID-19 pandemic found that respondents supported vaccination more when they are given the freedom to choose than when vaccination is made mandatory by the government (Schmelz, 2020; Schmelz & Bowles, 2021, 2022). Likewise, experimental evidence from Germany and the United States suggests that introducing a mandate elicited reactance when support for mandatory vaccination was low, even when the mandate was not self-relevant (Sprengholz, Felgendreff, et al., 2022). A higher level of psychological reactance elicited by mandating vaccination (vs. freedom to choose) in turn reduced intentions to get the COVID-19 vaccine (Sprengholz, Felgendreff, et al., 2022) as well as other vaccines (Sprengholz & Betsch, 2022; Sprengholz et al., 2021; Sprengholz, Felgendreff, et al., 2022). The two most cited causes of reactance are concerns about the safety of vaccines and the growing distrust of public authorities (Schmelz & Bowles, 2022; Slotte et al., 2022). Although none of these studies have examined vaccination uptake, evidence studying vaccination intentions suggest that the effectiveness of mandate policies may depend on people’s preexisting attitudes, such that they may indeed backfire for subpopulations and in regions that are strongly opposed to mandates, vaccines, and the public-health system.

Fortunately, evidence suggests a few strategies can be used to promote the effectiveness of vaccination mandates (Schmelz, 2020; Schmelz & Bowles, 2021, 2022). For example, making vaccines more easily accessible, communicating about the vaccine’s safety (Sprengholz, Korn, et al., 2022) and the benefits of high vaccination rates (Sprengholz & Betsch, 2020; Sprengholz, Felgendreff, et al., 2022), increasing trust in public institutions (Schmelz, 2020; Schmelz & Bowles, 2021), and reminding the public of precedent mandates for measles, mumps, rubella, and polio (Viskupicˇ et al., 2022) could prevent reactance and therefore minimize the potential detrimental effects of mandates among individuals who are strongly opposed to mandated vaccines. This stresses the importance of complementing policy-level interventions with well-designed persuasion strategies (Fishman et al., 2022; Schmelz & Bowles, 2022). The factors that were found to significantly moderate mandate policy effects are summarized in Table 1.

### Conclusion

What can we infer from prior evidence on mandate policies implemented to encourage vaccination? First, only a handful of studies have examined how mandates affect vaccination uptake, and the findings consistently showed that mandates were successful in increasing vaccination uptake, which in turn led to reductions in COVID-19 cases and deaths. However, these restrictions alone were insufficient to persuade hesitant individuals to vaccinate. Several reports found bipolar attitudes toward vaccination mandates, which is concerning given the possibility that mandating vaccines may lead to other undesirable behaviors (e.g., not wearing masks) aimed at restoring personal freedom.

The good news, however, is that vaccine hesitancy and negative attitudes toward mandates can be reduced by well-designed communication strategies about vaccine and vaccine mandates. Indeed, more behavioral investigations are needed to understand the competing theories of attitude change in response to policy implementation and what factors make a specific policy such as a vaccine mandate succeed or fail in a given setting.

## Effects of Vaccination Passports

Another strategy to promote COVID-19 vaccination was the introduction of vaccination passports, requiring everyone aged 12 years and older to present proof of vaccination to access a wide array of shared public spaces and events. Although such an option may appear less restrictive than mandates, these policies have raised similar concerns about potential violations of autonomy and freedom of choice and thus induce reactance. Studies that mostly focused on whether introducing vaccination passports increase vaccination rates at the population level were therefore conducted to address these concerns.

### Overall effects of vaccine passports on COVID-19 vaccination

As shown in Table 1, numerous studies, including experimental and correlational data analyses, indicate that vaccine passports could be an effective policy instrument. For example, in a cross-sectional study, Walkowiak et al. (2021) compared the vaccination outcomes in two neighboring countries, Lithuania and Poland, both of which implemented vaccination passports to some extent. Whereas Poland required having a vaccination certificate for international travel only, Lithuania imposed more coercive restrictions and limited individuals who failed to get vaccinated from accessing most services such as restaurants, sports facilities, and indoor events and even entering supermarkets or larger shops. Walkowiak et al. (2021) found that Lithuania’s strict policy led to markedly higher vaccination outcomes in all age groups than those of Poland.

A few quasi-experimental studies examined the causal impact of vaccine passports (vs. no passports) using variations in the timing of their implementation across regions and over time while controlling for other potential confounding variables across locations. First, Karaivanov et al. (2022) studied proof-of-vaccination requirements for accessing public venues and nonessential businesses across nine Canadian provinces in a difference-in-differences approach and found that the announcement of the policy had sizable and long-lasting impacts on the uptake of the first dose of COVID-19 vaccines. Likewise, two studies found similar effects in other countries. Mills and Rüttenauer (2022) analyzed the effects of vaccination-passport policy across Denmark, Israel, Italy, France, Germany, and Switzerland and found a significant increase in vaccination rates across all countries. Oliu-Barton et al. (2022) analyzed the effects of vaccination passports across three European countries—France, Germany, and Italy—and found a significant improvement in vaccination uptake across all countries. However, the magnitude of the impact varied significantly from one country to the other.

Such an effect was also found in an experimental setting. An experimental study of hypothetical vaccine passports that eases public-health restrictions such as travel, wearing face masks, and dining out at night among a representative sample of 5,000 Japanese adults was associated with an increase in vaccine acceptance (Okamoto et al., 2022).

### Heterogeneous effects across countries and subgroups

Although vaccination passports overall did not produce any negative effects, the magnitude of the change seemed to vary across regions. For instance, even within Canada, the effect ranged between 34% and 326% across the subregions. The larger increases were seen when there was a relatively short interval between the policy announcement and its implementation and when the prepolicy percentage of vaccinated was lower. Mills and Rüttenauer (2022) found no significant increase in total doses in Germany that already had a relatively high prepolicy vaccination coverage and in Denmark where the passport policy was introduced early, and thus there was insufficient vaccine supply. Across these European countries, the more unvaccinated people there were when the policy was introduced, the greater its effect was, consistent with Karaivanov et al.’s (2022) observations in Canada. Other potential cited factors for the regional variation are differences in the scope of the policy, the degree of political polarization, and the extent to which the policies are communicated by government or media (Karaivanov et al., 2022; Oliu-Barton et al., 2022). For example, in France and Italy, the implementation of the policy was supported by clear and consistent communications that the proof of vaccination is required to access most public places (Oliu-Barton et al., 2022).

Although studies examining the effects of vaccine passports in different countries suggest passport policy increased levels of vaccination, this increase was lower among the most vulnerable, or among those who were undecided or vaccine-hesitant (Okamoto et al., 2022; Ward et al., 2022). For example, Mills and Rüttenauer (2022) found that health passports most effectively promoted vaccination rates among younger groups. This is likely because older adults and low-income people do not engage much with the activities covered by the passport, such as eating out in restaurants or going to nightclubs.

Some studies have also warned about the polarizing effect of passports. For example, de Figueiredo et al. (2021) found that passports made those who already intend to get vaccinated even more positive, but the policy had the converse effect among those who had concerns about the vaccine, although this finding was correlational. The factors that were found to significantly moderate the passport-policy effects are summarized in Table 1.

### Conclusion

The large increases in vaccine uptake resulting from the implementation of vaccine passports are consistent with the evidence from analyzing vaccination mandates. Another important finding is that vaccine passports alone will not increase vaccine uptake among all groups. Studies in our review suggest that passports could be an important instrument for increasing uptake among certain subgroups such as younger people who generally do not perceive COVID-19 to be a serious threat to their personal health (Mills & Rüttenauer, 2022; Okamoto et al., 2022). We note, however, that our findings should be taken with caution given we could identify only a handful of studies that empirically tested the effects of vaccine passports. Indeed, unlike mandates and financial incentives, the vaccine passport is a relatively novel concept that was introduced during the global pandemic, and thus follow-up research is needed to evaluate its effects more critically in the event that similar policies are introduced in future pandemics.

## Effects of Financial Incentives

Many countries, including the United States, have offered financial incentives to vaccinate. Incentives are introduced to increase the attractiveness of vaccinating by providing an added material benefit. Similar to the criticisms cast on other COVID-19 vaccination policies, the implementation of incentive programs has also been widely debated for its potential to undermine voluntary support for vaccination and negatively affect motivation to vaccinate once these policies are lifted. Furthermore, because providing incentives at the population level is costly, numerous critics have questioned whether the public-health benefits gained from incentives outweigh the investments required to implement incentive programs. Given the controversy, laboratory experiments, field studies, and observational data analyses were conducted to examine the direction and magnitude of changes in vaccination rates induced by incentive structures.

### Overall effects of financial incentives on COVID-19 vaccination

Despite the criticisms about the provision of financial rewards, there is overall little evidence that incentive programs undermine vaccination. Indeed, most found studies that they either promote or at the least do not influence vaccination (see Table 1). For example, an analysis of COVID-19 vaccination before and after the lottery announcement in Ohio showed that the average number of COVID-19 vaccinations per day increased after the announcement, but this increase was more pronounced for low-income counties than high-income counties (Mallow et al., 2022). A 2-week pilot incentive program that guaranteed a $25 cash card in North Carolina also increased COVID-19 vaccination initiation or prevented decline in vaccination rates compared with other counties and states without such programs (Wong et al., 2022). Similar effects have been found in experimental settings. Specifically, an online experiment that asked American adults their intentions to vaccinate under different incentive conditions ($1,000, $1,500, $2,000) or under no incentive condition found that participants were 8% more likely to vaccinate when incentives were provided versus not (Robertson et al., 2021). Another experiment with American adults found that a financial incentive of $1,000 increased vaccination intentions up to 22.3% (Iyer et al., 2022). These findings were not restricted to American samples. A similar study with German participants found that intentions to vaccinate among previously unvaccinated individuals are higher when monetary incentives are provided than not (Sprengholz, Henkel, & Betsch, 2022). Among Swedish participants, offering a monetary incentive equivalent to $24 conditional on getting vaccinated increased vaccination rates by 4.7% from a baseline rate of 71.6%, indicating that incentives can promote vaccination even in countries with high vaccination rates (Campos-Mercade et al., 2021).

Some studies, however, found no effects of incentives. Specifically, a comparison of vaccination rates across states with or without incentive programs found that these states had similar trends in vaccination rates (Thirumurthy et al., 2022). A similar cross-state analysis of the effects of COVID-19 vaccine lotteries also found mixed results, with some states demonstrating a positive effect of lotteries on first-dose vaccinations and other states demonstrating a negative effect (Fuller et al., 2022). Randomly assigning unvaccinated members of a Medicaid managed-care health plan in California to different incentive structures to receive at least one dose of COVID-19 vaccines also yielded no change in vaccination rates (Jacobson et al., 2022). In fact, cross-state effects of financial lotteries diverge depending on whether the main outcome is first-dose vaccination or complete vaccinations (including the second dose). Specifically, lotteries positively affected first-dose vaccinations in most states that implemented the incentive policy, whereas the positive associations substantially reduced when considering complete vaccinations (Fuller et al., 2022). This suggests that the reward contingency may be an important determinant of the effectiveness of incentives. When lotteries are designed such that they incentivize only the first dose, they are insufficient in making people complete follow-up doses not directly incentivized.

### Incentive effects on subgroups

One important question is whether financial incentives are effective among groups that these incentives are primarily targeted at, such as those who are reluctant to vaccinate or those who lack financial resources and therefore could benefit more from incentive programs. Indeed, a survey on opinions about financial incentives found that 41% of participants reported cash as an important factor in getting them to vaccinate, and those with lower household income (less than $40,000) reported cash being more important than those with higher household income (Wong et al., 2022). Likewise, lotteries for COVID-19 vaccination were more effective in increasing vaccination rates in lower (vs. higher) income regions (Mallow et al., 2022). There is little evidence that financial incentives can motivate vaccination among hesitant individuals, however, because findings suggest that incentives yield a larger effect among undecided than hesitant individuals (Klüver et al., 2021). In fact, individuals who are most hesitant, those that underestimate the severity of COVID-19 and have little trust in the public-health system, report that no amount of monetary incentive can persuade them to vaccinate (Iyer et al., 2022). Furthermore, individuals who identify as Republicans, who tend to be higher in vaccine hesitancy than those who identify as Democrats (Fridman et al., 2021; Hornsey et al., 2020), are less responsive to financial incentives than the general population (Fishman et al., 2022; Robertson et al., 2021).

### Forms of incentives

Different forms of financial incentives can yield different outcomes, and one could expect that people would respond more positively to incentives that are larger, more probable, and more tangible. Indeed, several studies, most of which were conducted with non-U.S. samples, found that the willingness to get vaccinated increases with larger financial incentives, in which doubling the amount corresponds to more than doubling the effect on vaccination intentions (Klüver et al., 2021). Likewise, when asked of their intentions to vaccinate in scenarios that varied in incentive amount (from 0 to 5,000 euros in increments of 250 euros, and 10,000 euros), participants’ intentions increased with the payment amount (Sprengholz, Henkel, & Betsch, 2022). In contrast, studies conducted with U.S. samples found that participants were either nonresponsive to the changes in incentive amount (Fuller et al., 2022; Robertson et al., 2021) or that when directly asked about potential lottery structures preferred options that awarded less money to more people than more money to less people (Taber et al., 2021). For other aspects of incentives, people generally favor more probable and tangible ones. When previously unvaccinated participants were presented with different payment structures, such as a $1,000 cash reward, an equivalent tax penalty, a tax credit, and a larger amount in lotteries, vaccination intentions were statistically higher for $1,000 cash than other structures (Fishman et al., 2022). The factors that were found to significantly moderate incentive-policy effects are summarized in Table 1.

### Conclusion

Despite the lingering concerns about financial incentives potentially backfiring and thus undermining vaccination against COVID-19, the current evidence provides little support that they do. Numerous studies, including laboratory experiments, field studies, and observational data analyses, found that some positive amount of incentives can be effective in increasing intentions to vaccinate as well as actual vaccination. Of course, some found null results, suggesting that incentive policies are likely to be effective under the right conditions, such that they primarily target subpopulations that are more responsive to incentives and are strictly contingent on the type of behavior aimed to promote (e.g., first-dose vaccination or complete vaccination).

Nevertheless, the overall evidence also suggests that the positive impacts of financial-incentive policies are likely to be short term rather than long term. Specifically, results show that the provision of incentives is insufficient in changing people’s preexisting attitudes about vaccines. For example, even with the introduction of incentives, people’s willingness to vaccinate was strongly associated with their intentions prior to learning about the incentives (Taber et al., 2021), and people who are hesitant to vaccines were largely unresponsive to incentives (Iyer et al., 2022; Klüver et al., 2021). There is also some evidence that incentives make people consider vaccination as payment-contingent action because some report that they would not have vaccinated had they not been provided cash and that they intentionally delayed vaccination until they identified an event that provided incentives (Wong et al., 2022).

The above discussions stress the importance of complementing incentive policies with other provaccine policies and/or communication strategies that promote positive attitudes toward vaccination. For example, financial incentives are more effective in increasing intentions to vaccinate when they are coupled with communication strategies that emphasize personal freedom gained from vaccination and other measures that increased access to vaccination (e.g., the ability to get vaccines from local doctors; Klüver et al., 2021). It is also likely that incentives promote vaccination more when there is some level of consensus that vaccines are safe and effective and when people have some level of trust in the public-health system, all of which are important determinants of attitudes toward vaccines (Borah & Hwang, 2021; Kikut et al., 2022; Lohmann & Albarracín, 2022). Therefore, the design and implementation of incentive policies should accompany relevant public-health communications to maximize their effectiveness.

## Final Note

In this article, we reviewed empirical evidence on the effectiveness of three representative policies that were implemented worldwide to encourage COVID-19 vaccination: vaccination mandates, vaccination passports, and the provision of financial incentives. Relevant evidence on each of these policies leads to strikingly similar conclusions. First, unlike the widespread criticisms that government-imposed policies are likely to backfire because they undermine personal freedom and voluntary motivation to vaccinate, the evidence overall suggests that these policies can effectively increase vaccination at the population level.

Nonetheless, when we look more closely at their impacts on subpopulations, especially those that are strongly hesitant toward vaccines and distrust government and/or the public-health system, the policies themselves are insufficient in changing their preexisting attitudes on vaccines or their actual vaccination uptake. Hence, research suggests that one potential avenue to increase the impacts of vaccination policies on hesitant individuals would be to complement the policy-level interventions with well-designed public-health communications that are targeted at promoting positive attitudes on vaccines and the agencies implementing the policy. These may include introducing mandate policies in conjunction with persuasive messages that emphasize the social benefits of vaccine (Sprengholz, Felgendreff, et al., 2022). These may also include introducing incentive policies along with communications about enhanced freedom gained from vaccination (Klüver et al., 2021). Nonetheless, more research is needed that compares the effects of communication strategies in facilitating responsiveness to different types of policies.

The timing and structure of policies also determine their effectiveness. For example, policies are more effective when they are implemented shortly after their announcement and at earlier stages of the pandemic when vaccine coverage is still low and the majority is unvaccinated. This could indicate that policy interventions are less likely to be effective after people have formed their attitudes on vaccines and once vaccination has become politicized, as was the case in many countries. This also indicates that countries should design their policies well in advance of future epidemics and pandemics to intervene earlier rather than later. Likewise, as shown in our review of financial incentives, the policies and related public-health communications should be clear about what behavior they are aiming to promote (first-dose vaccination, complete vaccination) because there is little evidence that policy interventions produce a spillover effect; that is, the policies are unlikely to change behavior above and beyond those who are directly targeted by the policies.

Our review, however, is not without limitations. Specifically, because our review was restricted to policies implemented during the COVID-19 pandemic, it is unclear whether their effects are generalizable to policies relating to other infectious diseases (e.g., influenza). It is possible that the effects of COVID-19 vaccination policies were affected by other events that were particularly prominent during the pandemic, such as the prevalence of misinformation, political polarization, and unprecedented restrictions on individual and social behaviors. In this regard, one possible avenue for future research is to identify the key ingredients of policies that are effective in promoting vaccination against COVID-19 as well as other infectious diseases. In addition, although our review suggests that the effects of vaccination policies are positive or neutral at the population level, it also demonstrates that their effects can be heterogeneous, and there are numerous moderators that determine the level of their effectiveness on subpopulations and contexts (see Table 1). This emphasizes a strong need for future work to conduct meta-analyses that empirically test the impacts of these possible moderators to advance our understanding of the specific conditions and populations in which vaccination policies can yield the greatest effects. Likewise, future work can investigate whether the effects observed in the literature systematically differ according to the type of study designs (i.e., experimental, quasi-experimental, correlational) and outcome measures (vaccination intentions, vaccine uptake).

## Transparency

*Action Editor:* Norbert Schwarz

*Editor:* Interim Editorial Board

*Author Contributions*

B. Fayaz-Farkhad and H. Jung contributed equally and therefore should be considered joint first authors. Both authors approved the final manuscript for submission.
